# Absorption, Metabolism, and Excretion of [^14^C]-Tebipenem Pivoxil Hydrobromide (TBP-PI-HBr) in Healthy Male Subjects

**DOI:** 10.1128/aac.01509-22

**Published:** 2023-03-30

**Authors:** Vipul K. Gupta, Gary Maier, Leanne Gasink, Amanda Ek, Mary Fudeman, Praveen Srivastava, Angela Talley

**Affiliations:** a Spero Therapeutics, Inc., Cambridge, Massachusetts, USA; b Maier Metrics and Associates LLC, Worcester, Massachusetts, USA; c LBG Consulting, LLC, Wayne, Pennsylvania, USA; d Takeda Pharmaceuticals, Cambridge, Massachusetts, USA; e ImmunoGen, Inc., Waltham, Massachusetts, USA; f Nurix Therapeutics, Inc., San Francisco, California, USA

**Keywords:** absorption, excretion, mass balance, metabolism, tebipenem

## Abstract

Tebipenem pivoxil hydrobromide (TBP-PI-HBr) is an oral prodrug of pharmacologically active moiety tebipenem (TBP), which is a carbapenem with activity against multidrug-resistant Gram-negative pathogens. Conversion from the prodrug to the active moiety, namely, TBP, occurs in the enterocytes of the gastrointestinal tract via intestinal esterases. The absorption, metabolism, and excretion in humans were evaluated, following the administration of a single oral dose of [^14^C]-TBP-PI-HBr. Healthy male subjects (*n* = 8) received a single 600 mg oral dose of TBP-PI-HBr containing approximately 150 μCi of [^14^C]-TBP-PI-HBr. Blood, urine, and fecal samples were collected to determine the total radioactivity, concentrations of TBP (plasma only), and metabolite profiling and identification. The overall mean recovery of the total radioactivity in urine (38.7%) and feces (44.6%) combined was approximately 83.3% of the administered dose, with individual recoveries ranging from 80.1% to 85.0%. Plasma TBP LC-MS/MS and metabolite profiling data suggest that TBP was the main circulating component in plasma and that it accounts for approximately 54% of the total plasma radioactivity, based on the plasma AUC ratio of TBP/total radioactivity. The ring-open metabolite LJC 11562 was another major component in plasma (>10%). TBP (M12), LJC 11562, and four trace to minor metabolites were identified/characterized in the urine. TBP-PI, TBP (M12), and 11 trace to minor metabolites were identified/characterized in the feces. The renal and fecal routes are major clearance pathways in the elimination of [^14^C]-TBP-PI-HBr, with a mean combined recovery of 83.3%. TBP and its inactive ring-open metabolite LJC 11562 were the major circulating metabolites in the plasma.

## INTRODUCTION

**FIG 1:**
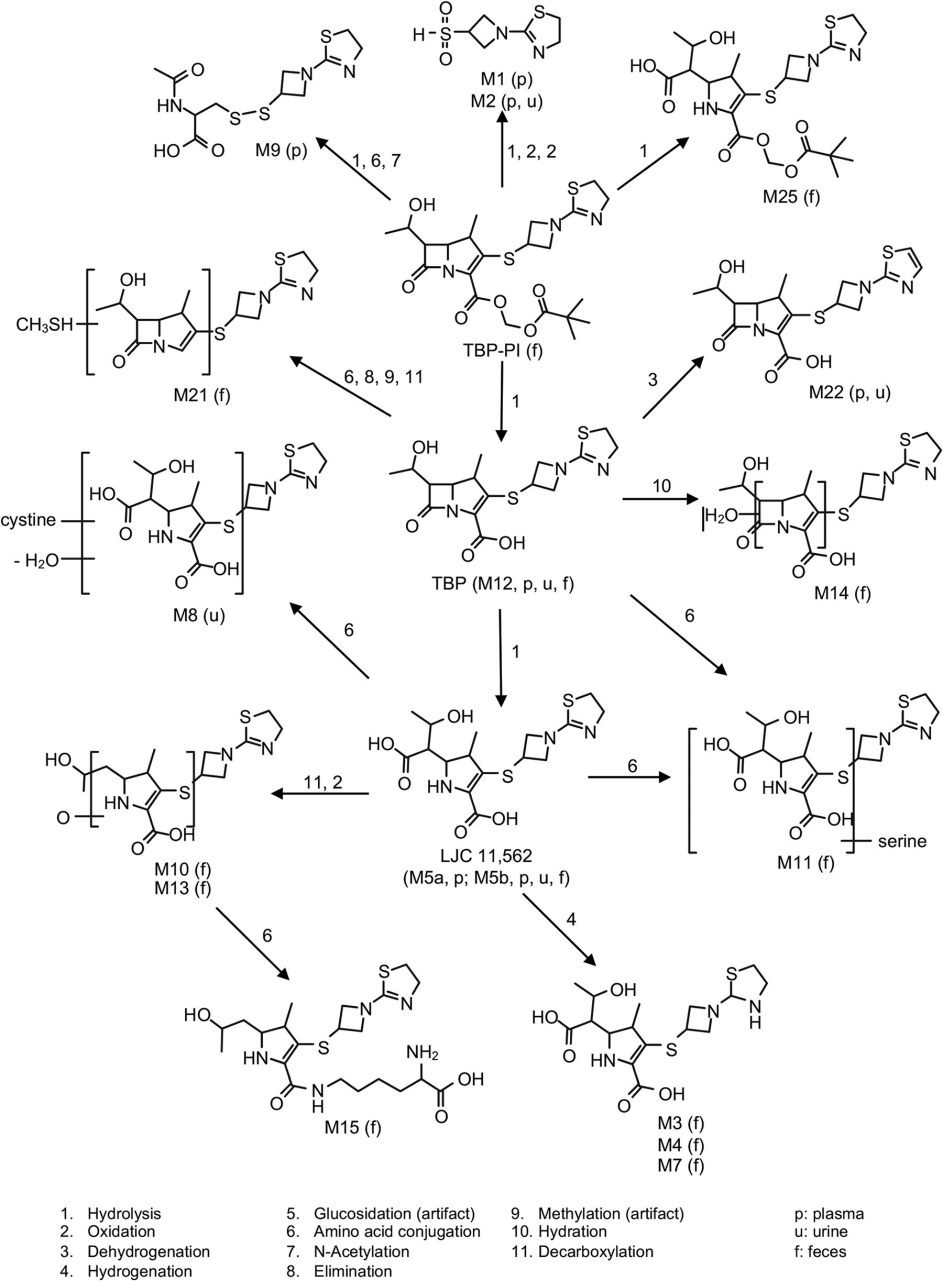
Structure of TBP and metabolites and proposed biotransformation pathway.

Tebipenem pivoxil hydrobromide (TBP-PI-HBr) is an oral prodrug of active moiety tebipenem (TBP), and it aids in the improved absorption and bioavailability of TBP (Fig. 1). Conversion from TBP-PI-HBr to TBP, the pharmacologically active moiety, reportedly occurs in enterocytes of the gastrointestinal tract via intestinal esterases (1). TBP-PI-HBr is the first oral carbapenem, and it is being developed for the treatment of serious infections, including complicated urinary tract infections and acute pyelonephritis. TBP exhibits potent *in vitro* activity against multidrug-resistant (MDR) pathogens, including fluoroquinolone-resistant and extended-spectrum β-lactamase (ESBL)-producing Enterobacterales (2–6). TBP has demonstrated *in vivo* activity in neutropenic murine thigh infection models and in lung infection models (7). In human single-ascending and multiple-ascending dose studies, the PK profile of TBP was dose-proportional after single TBP-PI-HBr doses of 100 to 900 mg as well as after multiple daily doses of 300 and 600 mg every 8 h. The peak concentrations (Cmax) of TBP were reached within 2 h, and the elimination half-life (t_1/2_) was approximately 1 h. TBP plasma exposure (AUC) was comparable during fed and fasted states, and no accumulation was observed after dosing for 14 days (8).

Information about the metabolism and excretion of new drugs is important to determine. The metabolism of a new drug should be determined to minimize concerns about potential toxicity from major metabolites, which are metabolites with a systemic exposure that is >10% of the total exposure. Excretion pathways are important to investigate to determine the safety for use in patients with impaired renal or hepatic function.

Nonclinical studies with TBP-PI or TBP-PI-HBr indicated rapid absorption after oral administration with TBP, with bioavailability up to 71.4%. At least 90% of the TBP-PI radioactive dose was recovered in the urine or feces within 48 h in rats and monkeys (data on file, Spero Therapeutics). Tissue distribution was extensive, and TBP was the primary metabolite of TBP-PI. This study evaluated the absorption, metabolism, and excretion of TBP-PI-HBr, following the administration of a single oral dose of [^14^C]-TBP-PI-HBr to healthy males, and it also characterized the metabolites that were present in the plasma, urine, and feces.

## RESULTS

Eight subjects were enrolled, and all were included in the safety and PK analyses (Table 1). Males were aged 23 to 54 years with a BMI of 22.0 to 31.4 kg/m^2^. Six subjects (75.0%) were white, and two subjects (25.0%) were Black or African-American.

**TABLE 1:** Baseline characteristics

Characteristic	TBP-PI-HBr 600 mg [^14^C] (*n* = 8)
Age, years^*a*^	38.4 ± 11.4
Male, n (%)	8 (100)
Race, n (%)	
White	6 (75)
Black or African-American	2 (25)
Height, cm^*a*^	176.7 ± 7.5
Weight, kg^*a*^	83.0 ± 13.3
Body mass index, kg/m^2^^*a*^	26.5 ± 3.2

a Mean ± standard deviation.

### Pharmacokinetics.

**FIG 2:**
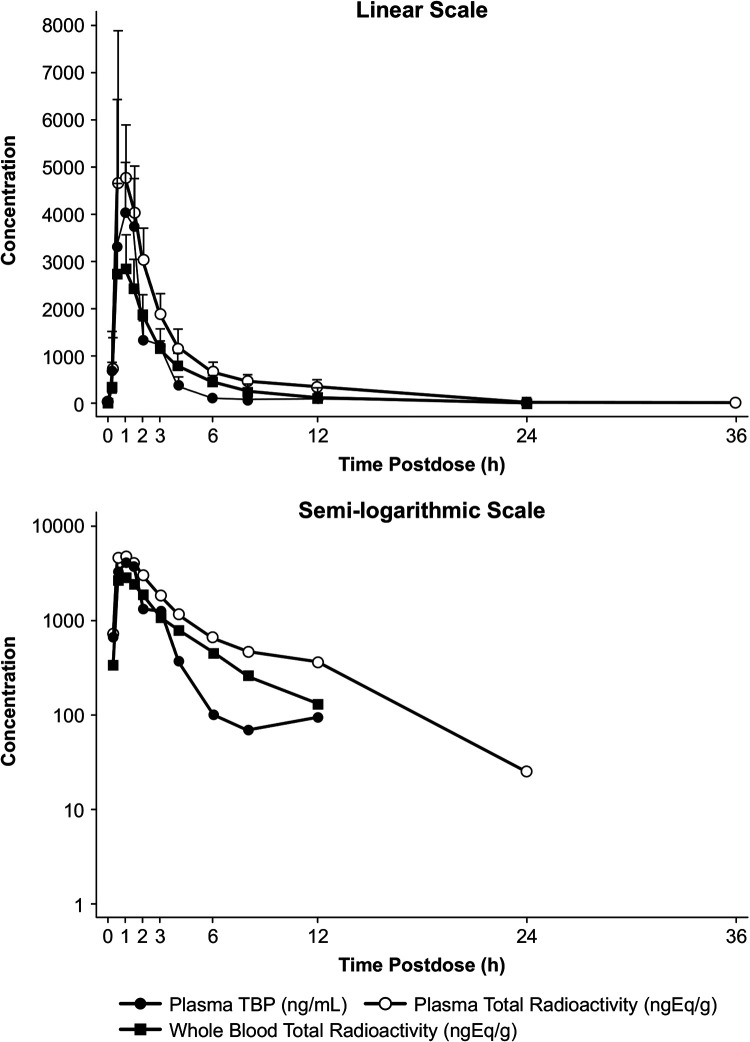
Arithmetic mean plasma concentrations of TBP and total radioactivity in plasma and whole blood, following a single oral dose of TBP-PI-HBr (linear and semilogarithmic scales). A 600 mg dose of TBP-PI-HBr contained approximately 150 μCi of [14C]-TBP-PI-HBr.

Following the oral administration of [^14^C]-TBP-PI-HBr, the PK profile for TBP was characterized via rapid absorption in the systemic circulation, with a median T_max_ value of 1.0 h (range: 0.5 to 1.5 h) in the plasma (Table 2). After reaching Cmax, the TBP plasma concentrations declined in a biphasic manner that was indicative of the likely distribution of TBP into tissues (Fig. 2). The TBP half-life could not be calculated, based on pre-specified criteria (adjusted R^2^ was <0.7 for 7 subjects); however, the TBP concentrations fell below the limit of quantification (<0.0072 mg/mL) 12 h following the administration of [^14^C]-TBP-PI-HBr, indicating a relatively short half-life. TBP has a reported half-life of approximately 1 h, using an IR TBP-PI-HBr tablet in healthy subjects (8).

**TABLE 2:** Summary of pharmacokinetic parameters for TBP in plasma and total radioactivity in plasma and whole blood^*a*^

Parameter	Estimated plasma TBP	Plasma total radioactivity	Whole blood total radioactivity
AUC_0-inf_ (h·ng/mL)^*b*^	Not calculated	18,500 (28.0) [7]	8,850 (32.9) [6]
AUC%_extrap_, (%)	Not calculated	12.1 (22.3) [7]	13.4 (11.0) [6]
AUC_0-last_ (h·ng/mL)^*b*^	8,340 (25.0) [8]	15,500 (28.6) [8]	8,570 (35.9) [8]
Cmax, (ng/mL)^*b*^	4,540 (46.2) [8]	5,450 (38.9) [8]	3,150 (43.2) [8]
T_max_ (h)	1.0 (0.5 to 1.5) [8]	1.0 (0.5 to 1.5) [8]	1.0 (0.5 to 1.5) [8]
T_last_ (h)	12 (12 to 24) [8]	12 (12 to 24) [8]	8 (6 to 12) [8]
Half-life (h)	Not calculated	5.98 (55.3) [8]	3.52 (54.7) [8]
AUC_0-inf_ plasma TBP/total radioactivity ratio	Not applicable	Not calculated	Not applicable
AUC_0-last_ plasma TBP/total radioactivity ratio	Not applicable	0.536 (6.3) [8]	Not applicable
AUC_0-inf_, blood/plasma ratio	Not applicable	Not applicable	0.566 (10.6) [5]
AUC_0-last_, blood/plasma ratio	Not applicable	Not applicable	0.551 (10.6) [8]

a Values are geometric means (CV%) [n]. T_max_ and T_last_ are medians (min to max) [n]. Not applicable indicates that the value was not determined for the sample. Not calculated indicates that the value was not calculated because the values did not meet the prespecified calculation criteria (due to the adjusted R^2^ being <0.7 for 7 subjects).

b The units for the total radioactivity of AUC and Cmax are h·ngEq/g and ngEq/g, respectively.

The maximal levels (Cmax) of the total radioactivity in the plasma and whole blood were reached at the same time as for TBP, with a median t_max_ of 1.0 h (Table 2). The levels of total radioactivity in the plasma declined in a biphasic manner, with a geometric mean t_1/2_ of 6.0 h (range: 3.2 to 16.6 h). The levels of total radioactivity in the plasma were quantifiable for up to 12 h post-dose in all subjects and 24 h post-dose in 1 subject. The whole blood total radioactivity declined slightly more rapidly than did the plasma total radioactivity, with a geometric mean t_1/2_ of 3.5 h (range: 1.8 to 8.4 h). The levels of total radioactivity in the whole blood were quantifiable for up to 6 h post dose in all subjects, up to 8 h post dose in 6 subjects, and up to 12 h post dose in 3 subjects.

The geometric mean AUC_0-last_ plasma TBP/total radioactivity ratio was 0.536, suggesting that metabolites contribute toward the remaining circulating total radioactivity in the plasma. The geometric mean whole blood/plasma AUC_0-last_ ratio for the total radioactivity was approximately 0.55, indicating a low association of TBP-PI-HBr radioactivity with cellular components.

### Mass balance.

**FIG 3:**
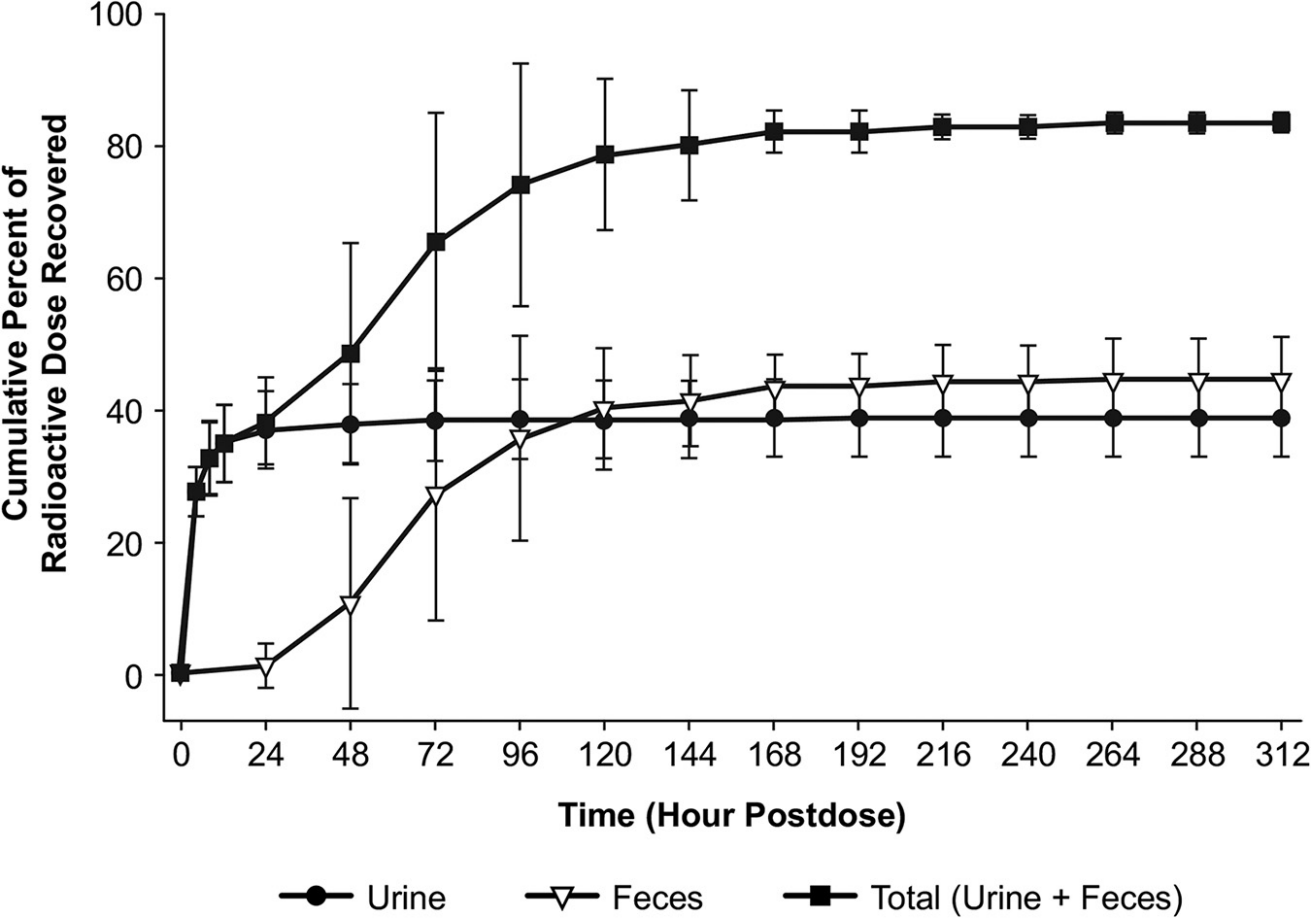
Arithmetic mean (±SD) cumulative percentage of the radioactive dose that was recovered in feces at specified intervals after a single 600 mg (150 μCi) oral dose of [^14^C]-TBP-PI-HBr.

The mean recovery of radioactivity in urine and feces combined was approximately 83.3% of the administered radioactive dose over the 312 h study period (Fig. 3). The mean recovery of total radioactivity in the urine and feces was 38.7% and 44.6%, respectively. Most of the administered radioactivity was recovered in the first 144 h post-dose in the urine and feces combined (80.0%). Excretion in the urine was the highest during the 0 to 4 h post-dose interval, whereas fecal excretion was highest during the 48 to 72 h post-dose interval. The levels of radioactivity in the feces fell BLQ (<0.0072 mg/mL) between 96 to 240 h for all subjects.

### Metabolite profiling.

Metabolite profiling and identification in the plasma, urine, and feces indicated that TBP-PI underwent rapid and extensive hydrolysis at the pivalate ester, following a single oral dose of [^14^C]-TBP-PI-HBr (Table 3). Intact TBP-PI was not detected in the plasma and accounted for only 0.58% of the total radioactive dose in the feces. TBP-PI underwent rapid and extensive hydrolysis at the pivalate ester in healthy male subjects after a single oral dose of [14C]-TBP-PI-HBr to yield TBP as the putative major circulating component, based primarily upon the AUC ratio of TBP to total radioactivity.

**TABLE 3:** Summary of the radiolabeled components quantified and identified in plasma, urine, and feces^*a*^

Component designation	Retention time (minutes)	Proposed identification	Matrix^*b*^		
			Plasma	Urine	Feces
M1	3.00	HSADT	X		
M2	6.33 to 9.17	HSADT	X	X	
M3	12.67 to 12.83	Dihydro-LJC 11562			X
M4	13.00	Dihydro-LJC 11562			X
M5a (LJC 11562)	16.67 to 17.17	LJC 11562	X		
M6	18.83	Unknown		X	
M5b (LJC 11562)	18.83 to 19.50	LJC 11562	X	X	X
M7	19.50 to 20.17	Dihydro-LJC 11562			X
M8	21.83 to 22.00	Cystinyl-LJC 11562		X	
M9	26.33 to 26.50	MADT mercapturic acid	X		
M10	26.67 to 27.33	Descarboxyl-oxy-LJC 11562			X
M11	27.50 to 27.83	Serinyl-LJC 11562			X
M12 (TBP)	28.50 to 29.33	TBP	X	X	X
M13	29.33 to 30.00	Descarboxyl-oxy-LJC 11562			X
M14	30.17 to 30.33	Dihydro-oxy-TBP			X
M15	30.50 to 31.00	Descarboxyl-lysinyl-LJC 11562			X
M16	31.17 to 31.33	Unknown			X
M17	32.17 to 32.33	LJC 11562 glucose	X		
M18	33.00 to 33.17	LJC 11562 glucose	X		
M19	36.67 to 36.83	Methyl-LJC 11562	X		
M20	38.33 to 38.50	Methyl-LJC 11562	X		X
M21	38.67 to 39.17	Descarboxyl-TBP-methylsulfide			X
M22	39.17 to 39.33	Dehydro-TBP	X	X	
M23	39.17 to 39.33	Unknown			X
M24	39.50 to 39.83	Unknown	X		X
M25	40.17 to 40.50	Dihydro-oxy-TBP-PI			X
M26	40.50 to 40.67	Unknown			X
TBP-PI	43.50	TBP-PI			X
M27	44.00	Methyl-dihydro-oxy-TBP-PI			X

a Retention time ranges are from profiling analyses of all matrices. HSADT, hydrosulfonyl-azetidinyl-dihydrothiazole; MADT, mercapto-azetidinyl-dihydrothiazole.

b The component is found in the matrix designated with “X”.

The totality of the plasma and urine data combined with the TBP-fortified control plasma data indicated that TBP represents the major circulating component in the plasma. Other minor components detected in the plasma included LJC 11562-glucose, hydrosulfonyl-azetidinyldihydrothiazole (HSADT), mercapto-azetidinyl-dihydrothiazole, dehydro-TBP, and one unknown metabolite.

**FIG 4:**
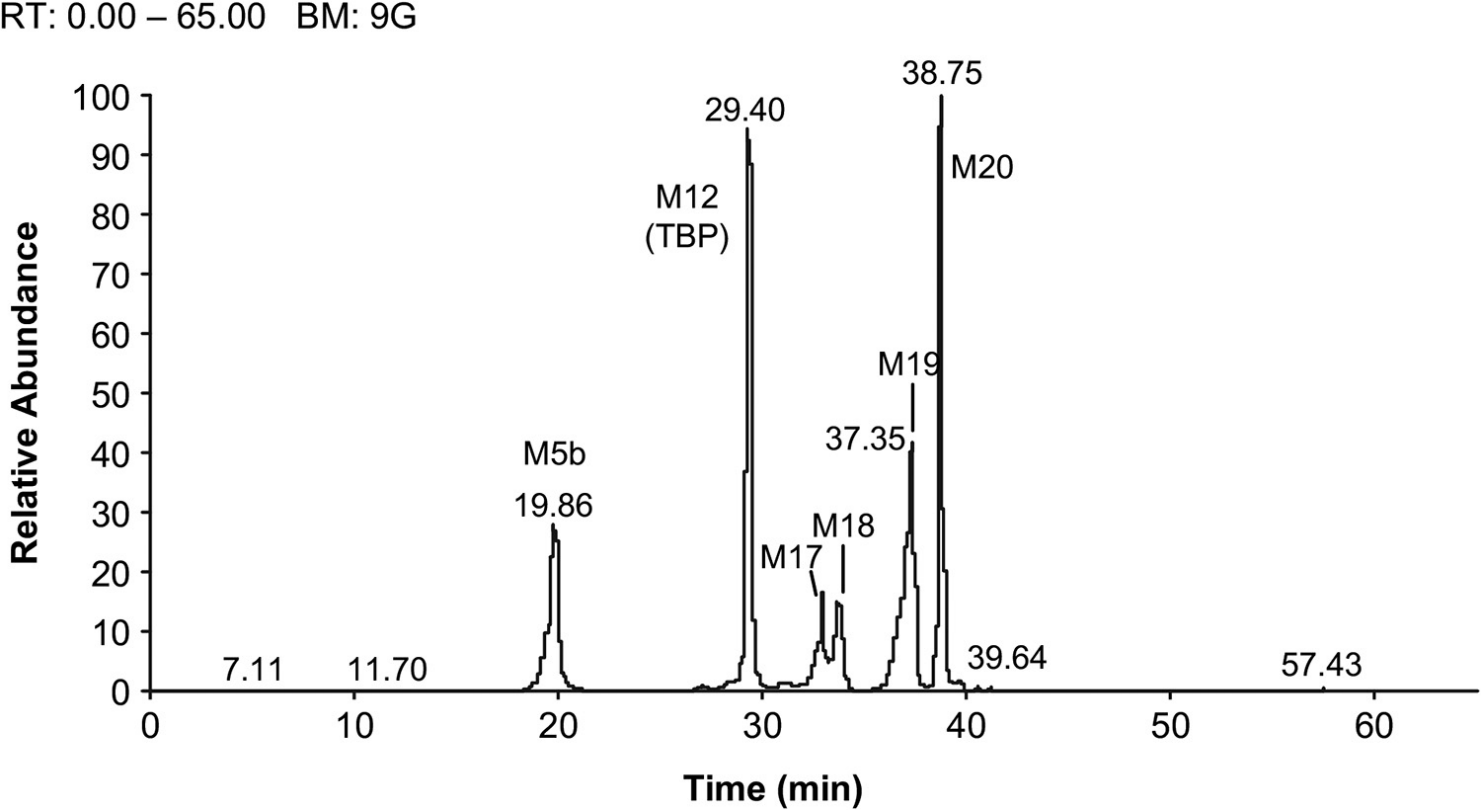
Radiochromatogram from blank plasma spiked with TBP and an analysis of plasma and feces samples after a single oral dose of [14C]-TBP-PI-HBr to male human subjects.

**FIG 5:**
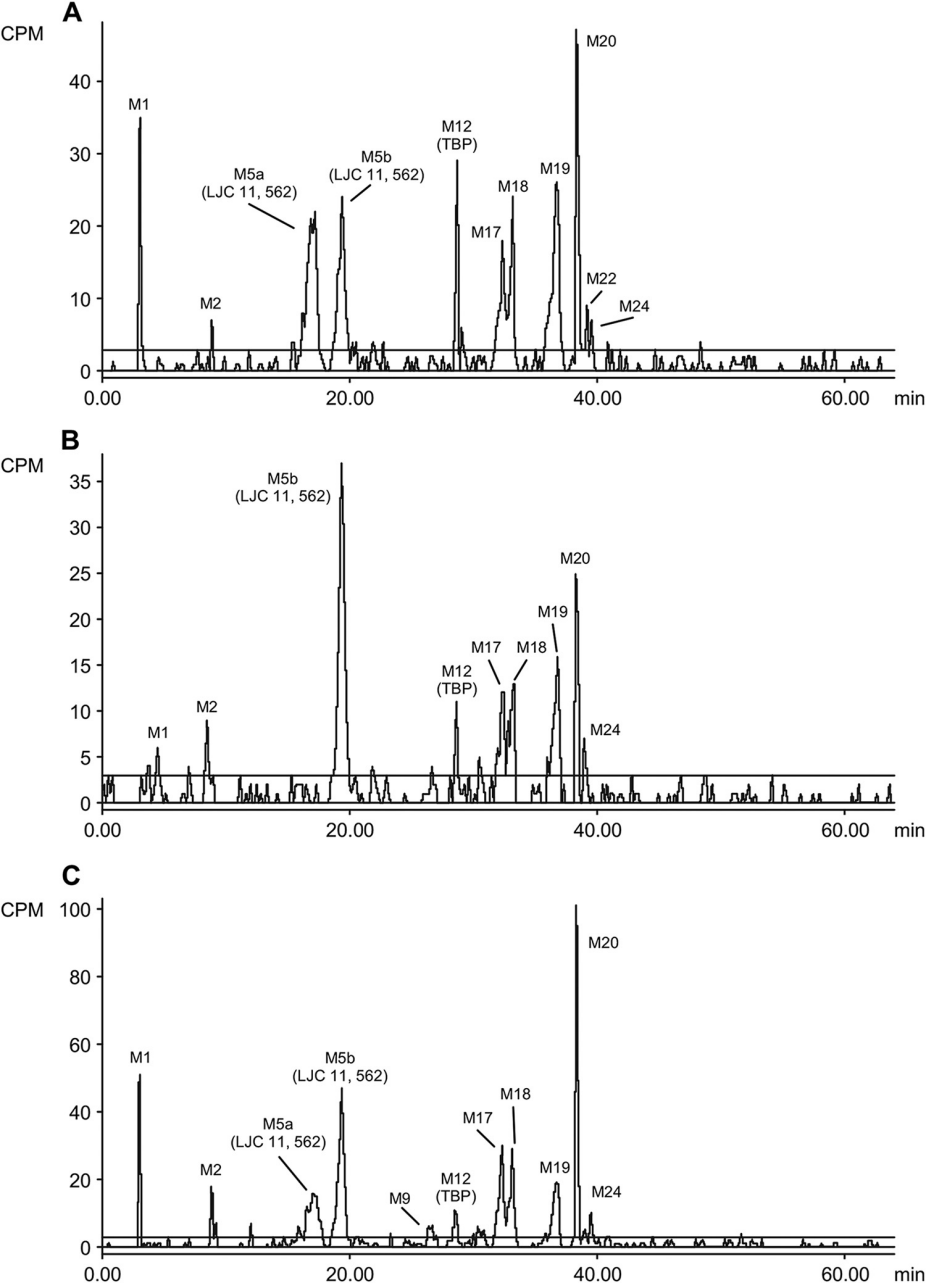
Radiochromatograms from an analysis of 0.25 h to 6 h AUC-pooled plasma samples after a single oral dose of [^14^C]-TBP-PI-HBr to male human subjects (600 mg/subject, 150 μCi/subject).

**FIG 6:**
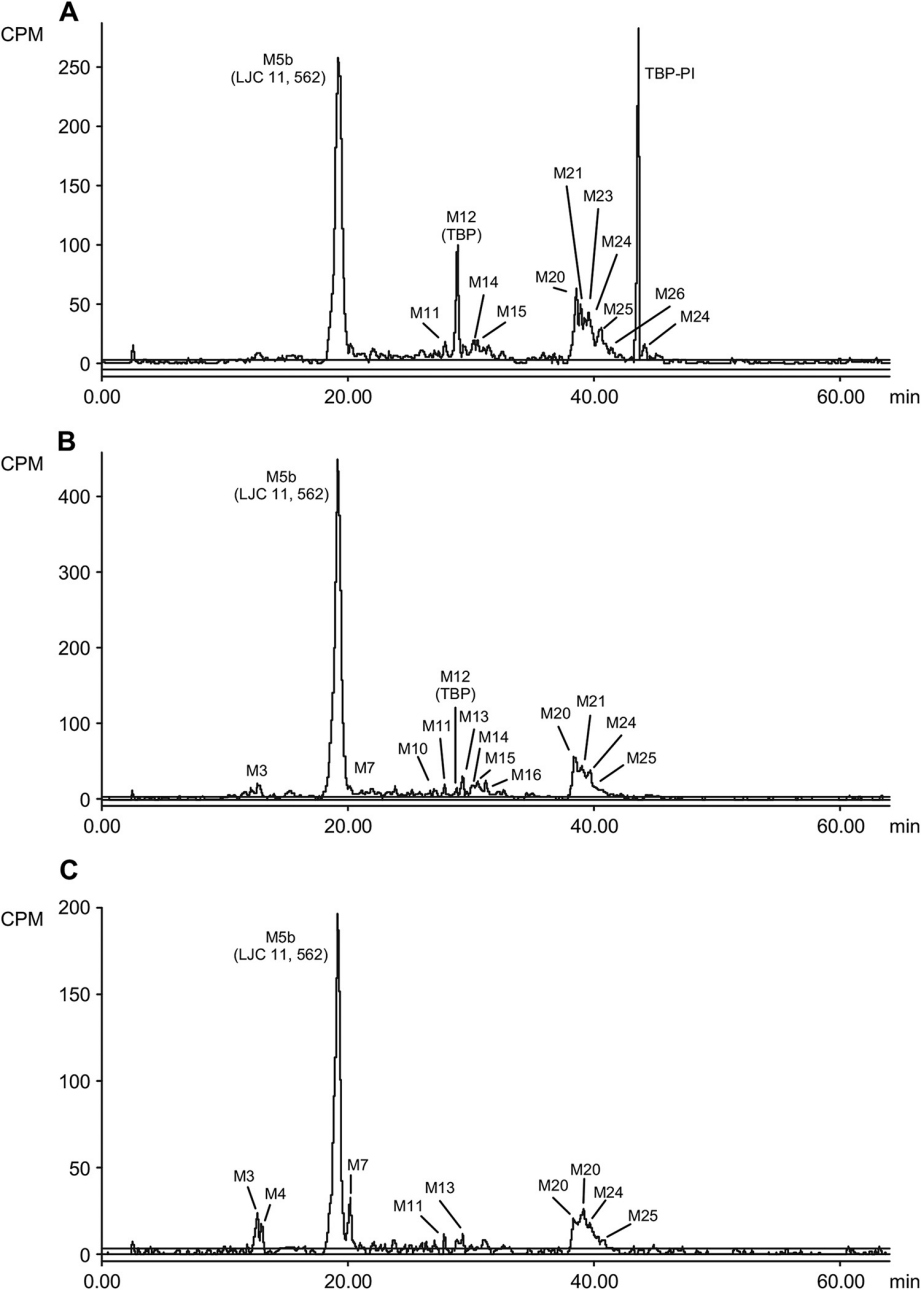
Radiochromatograms from an analysis of fecal samples after a single oral dose of [^14^C]-TBP-PI-HBr to male human subjects.

TBP was a major component in the urine from all subjects, and it accounted for 29.6% of the total radioactive dose or approximately 80% of the radioactivity in the urine. LJC 11562 was a minor metabolite in urine, accounting for 5.27% of the radioactive dose, whereas HSADT, cystinyl-LJC 11562, dehydro-TBP, and an unknown metabolite were detected as trace metabolites that each accounted for less than 1% of the total dose. TBP and 11 metabolites were identified/characterized in feces. TBP accounted for 0.308% of the total radioactive dose, whereas LJC 11562 was the major putative component in feces and represented 16.6% of the total radioactive dose. All other metabolites represented less than 15% to 20% of the total administered dose. Radiochromatograms are shown in Fig. 4, 5, and 6.

### Safety/tolerability.

Treatment-emergent adverse events (TEAEs) were reported in 3 (37.5%) subjects. All TEAEs were mild in severity. The only TEAEs were diarrhea occurring in two (25%) subjects, ear pain occurring in one subject (12.5%), and pollakiuria occurring in one subject (12.5%). Both cases of diarrhea were considered to be related to therapy. No deaths, serious adverse events, or TEAEs leading to discontinuation occurred. No clinically significant abnormalities for clinical laboratory testing, ECG, vital signs, or physical examination were reported.

## DISCUSSION

This phase 1, open-label, nonrandomized, single-dose study in healthy male subjects was designed to determine the absorption, metabolism, and excretion of [^14^C]-TBP-PI-HBr and to characterize and determine the metabolites present in the plasma, urine, and feces following a single, oral, 600 mg dose of TBP-PI-HBr containing approximately 150 μCi of [^14^C]-TBP-PI-HBr.

The radioactive dose of approximately 150 μCi was expected to provide a sufficient radioactive signal to achieve the study objectives with minimal radiation risk to the subjects. Dosimetry analyses of data from male pigmented rats indicated that the overall whole-body radiation dose in a male subject would be 0.976 mrem (0.00976 mSv), following the administration of a single 150-μCi (5.55 MBq) oral dose of [^14^C]-TBP-PI-HBr. This value is well below the FDA exposure limit of 3,000 mrem (30 mSv) for radioactive drugs to be used for absorption, distribution, metabolism, and excretion research. Based on the PK and dosimetry data, the administration of a single 150-μCi (5.55 MBq) oral dose of [^14^C]-TBP-PI-HBr was not expected to represent a significant radiation exposure risk to human male subjects. As TBP is already approved in Japan for a pediatric population (as a TBP-PI prodrug) and the disposition of the prodrug (either as TBP-PI or TBP-PI-HBr) and active moiety (TBP) have been adequately characterized across many studies, we did not consider absolute bioavailability determination or, hence, a micro tracer dosing approach for this study. Further, a population PK analysis was conducted with TBP-PI-HBr, and no difference was found in PK parameters between males and females (9).

LJC 11562 (M5a/M5b/M19/M20) and LJC 11562 glucose (M17 and M18) were major components in plasma, accounting for 56.3% and 13.2% of the total plasma radioactivity exposure, respectively. Apparently, TBP was minor and accounted for 4.02% of the total plasma radioactivity exposure. However, based on TBP-fortified control plasma data, the percentages of LJC 11562 (M5a/M5b/M19/M20) and LJC 11562 glucose (M17 and M18) in plasma are probable artifacts of sample processing. Looking across plasma TBP exposure together with plasma sample processing artifacts for metabolite profiling, the totality of the data suggests that TBP was the main circulating component in the plasma. Plasma TBP LC-MS/MS and metabolite profiling data suggest that TBP was the main circulating component in the plasma and accounts for approximately 54% of the total plasma radioactivity, based on the plasma AUC_0-last_ ratio of TBP/total radioactivity. The ring-open inactive metabolite LJC 11562 was another major component in the plasma (>10%). All other metabolites in the plasma were considered to be minor (<10%). Metabolite profiling and identification in the plasma, urine, and feces indicated that TBP-PI underwent rapid and extensive hydrolysis at the pivalate ester in healthy male subjects, following a single oral dose of [^14^C]-TBP-PI-HBr, to yield TBP as the major component in the plasma. Intact prodrug (TBP-PI) was not detected in the plasma and accounted for only 0.58% of the total radioactive dose in the feces. The pharmacologically inactive ring-open metabolite LJC 11562 was found in the plasma (>10%) and in the feces (16.6% of the total radioactive dose) as a secondary metabolite. The hydrolysis of the β-lactam ring either chemically or enzymatically results in inactive compound, and it is commonly observed across members of the carbapenem class, such as ertapenem, meropenem, and doripenem (1, 10). Numerous minor (<10%) metabolites were also observed in the plasma and in the feces.

A 600 mg dose of TBP-PI-HBr was selected for use in this study, as this is considered to be therapeutically relevant and consistent with the dose that was evaluated in a phase 3 study of patients with complicated urinary tract infections (cUTI) (11). The 600 mg dose was selected as the dose that was the most likely to achieve sufficient plasma exposures for efficacy against target cUTI pathogens in clinical settings. This dose was previously demonstrated to be well-tolerated in healthy adult subjects in a single-ascending and a multiple-ascending dose study (8). The PK profile of TBP that was obtained with a 600 mg oral dose in the single-ascending dose phase was similar to the PK profile reported in this study, with a half-life of 1.1 h, T_max_ of 1.3 h, and Cmax of 6,203 ng/mL.

The median t_max_ for TBP was observed to be 1.0 h, indicating a rapid conversion of TBP-PI-HBr to TBP. Conversion from the prodrug to the pharmacologically active moiety, TBP, is reported to occur in the enterocytes of the gastrointestinal tract via intestinal esterases (12). The rapid T_max_ observation in the current study is consistent with that reported in a previous study of healthy subjects, in which TBP exhibited a similar T_max_, following the administration of TBP-PI-HBr (8). The geometric mean whole blood/plasma AUC_0-last_ ratio for the total radioactivity was 0.55, indicating a low association of TBP-PI-HBr radioactivity with red blood cells.

The overall mean recovery of radioactivity in the urine and feces was approximately 83.3% of the administered radioactive dose over the 312 h study period, with a mean recovery of total radioactivity of 38.7% and 44.6% for feces. In healthy subjects, the TBP recovery in urine was unchanged, and the percentage of the administered dose is reported to be 55% to 60% (8). The results in the present study are lower than those that were observed in a previous study in healthy subjects; however, these results are similar to the results that were observed with mass balance studies in rats and monkeys, in which radioactivity was excreted approximately equally in feces and urine, following the oral administration of [^14^C]-TBP-PI (13). Taken together, these results suggest the roles of the renal and fecal routes in the elimination of [^14^C]-TBP-PI-HBr in humans.

6 of the 8 subjects that were dosed in this study achieved a mass balance recovery ranging from 80.1% to 85.0%. which was slightly lower than the targeted mass balance recovery of ≥90% at discharge. However, <1% of the radioactivity was recovered in two consecutive sample collection intervals for these subjects at later time points. Thus, the >80% total recovery that was obtained in this study was considered to be adequate to meet the objectives of the study.

Knowledge of the metabolism and excretion of a parent drug and its metabolites is useful for evaluating the Metabolites in Safety Testing requirements that are enumerated in the Food and Drug Administration (FDA) Guidance (14) and in the guidance of other regulatory authorities (ICH 1997) (15) as well as for elucidating the likelihood of effects of renal or hepatic impairment on the disposition of TBP-PI-HBr. The results from this study are useful in the characterization of the disposition of TBP-PI-HBr in humans and indicated no to negligible levels of the prodrug in the plasma and feces. The major (>10% of total radioactivity) circulating metabolites in plasma are considered to be the active moiety TBP and the inactive ring open metabolite LJC-11562. TBP was a major component in the urine from all subjects, and it accounted for 29.6% of the total radioactive dose (approximately 80% of radioactivity) in the urine. LJC 11562 was a minor metabolite in the urine, accounting for 5.27% of the radioactive dose. TBP and 11 metabolites were identified/characterized in the feces. TBP accounted for 0.308% of the total radioactive dose, whereas LJC 11562 was the major putative component in the feces, representing 16.6% of the total radioactive dose.

The single, oral, 600 mg dose of [^14^C]-TBP-PI-HBr was well-tolerated. Only four TEAEs were reported by three subjects, of which two events of diarrhea were considered to be possibly related to [^14^C]-TBP-PI-HBr. All of the TEAEs were mild in severity, and no TEAEs leading to study discontinuation, no serious AEs, and no deaths were reported. Further, no clinically significant findings were observed for clinical laboratory evaluations, vital signs, physical examinations, or ECG parameters.

In summary, this study demonstrated that TBP-PI-HBr was rapidly converted to TBP and absorbed in the systemic circulation, with a median TBP t_max_ of 1.0 h in the plasma. The total radioactivity in both the plasma and the whole blood decreased rapidly. A low association of total radioactivity with the cellular components of blood was observed, based on the geometric mean blood to plasma ratio being 0.551. The TBP plasma to total radioactivity ratio of 0.536 indicated that other metabolites contributed to the total radioactivity in the plasma. The cumulative mean recovery of radioactivity from the urine and the feces suggested that both renal and fecal routes are important for the elimination of TBP. Metabolite profiling results indicated no intact prodrug in plasma, whereas TBP and its inactive ring open metabolite LJC 11562 were considered to be the major circulating metabolites.

## MATERIALS AND METHODS

The study was conducted in accordance with the U.S. Code of Federal Regulations and the ethical principles of the Declaration of Helsinki, Good Clinical Practices, and the guidelines of the International Council for Harmonisation. The study protocol and all amendments were reviewed and approved by an Institutional Review Board for the study center. Informed consent was obtained from each subject in writing before randomization.

### Study design.

This was a Phase 1, open-label, single-dose study. The objectives were: to determine the mass balance, routes of elimination, and metabolite profiles and structures; to assess the total radioactivity PK in the whole blood and the plasma, as well as the TBP PK (plasma only); and to assess the safety/tolerability, following a single dose of 600 mg TBP-PI-HBr (containing approximately 150 μCi of [^14^C]-TBP-PI-HBr). Male subjects were screened within 28 days of the administration of the study drug, and eligible subjects were confined to an inpatient clinical research unit, beginning 1 day prior to dosing. On the morning of day 1, each of the 8 subjects received a single, oral, 600 mg dose of TBP-PI-HBr. The study drug was provided as hand-filled capsules containing a blended powder of radiolabeled and nonradiolabeled active pharmaceutical ingredients, such that 3 capsules, taken with 240 mL of room-temperature water, provided the target dose of 600 mg TBP-PI-HBr containing approximately 150 μCi of [^14^C]-TBP-PI-HBr. All subjects fasted overnight for at least 10 h and refrained from consuming water for 1 h prior to dosing until 2 h post-dose, excluding the amount of water consumed at dosing, and they continued fasting until 2 h post-dose.

The subjects were confined to the inpatient unit until at least day 5, and they were individually discharged on day 5 if they had ≥90% mass balance recovery and if ≤1% of the total radioactive dose was recovered in the feces in 2 consecutive 24 h periods in which fecal samples were obtained. If these criteria were not met by day 5, the subjects remained confined to the inpatient unit until all of the discharge criteria were met, up to a maximum of 14 days, to continue blood sampling in the blood and in the feces.

### Subject selection.

Adult men, 18 to 55 years of age, inclusive, were eligible if they had a body mass index (BMI) of ≥18.0 and ≤32.0 kg/m^2^, had a body weight between 50 and 100 kg, and were medically healthy without clinically significant findings regarding their medical history, physical examination, vital signs, 12-lead electrocardiogram (ECG), hematology, biochemistry, coagulation, and urinalysis. Subjects were excluded for any clinically significant medical condition at screening or baseline, history of any clinically significant allergic disease or hypersensitivity, or any medical condition or laboratory abnormality that could interfere with the conduct of the study. Also excluded were those with a history of drug or alcohol abuse within 2 years, alcohol consumption of >21 units per week, tobacco, nicotine, or nicotine-replacement product use within 30 days of baseline, or a positive urine drug screen or alcohol breath test at baseline.

### Study assessments.

Study assessments performed at screening, baseline, and discharge included complete physical examinations, vital signs (blood pressure, heart rate, respiratory rate, oral temperature), 12-lead ECG, clinical laboratory tests (hematology, chemistry, coagulation, and urinalysis), and the monitoring of adverse events (AEs). In addition, subjects had a urine drug screen performed at screening and at baseline, as well as an alcohol breath test performed at baseline.

Blood samples were collected to determine the TBP concentrations (whole blood), total radioactivity (whole blood and plasma), and metabolite profiling and identification (plasma) at the following time points: pre-dose (0), at 0.25, 0.5, 1, 1.5, 2, 3, 4, 6, 8, 12, 24, 36, 48, 72, and 96 h post-dose on days 1 to 5, and additionally for TBP concentrations (whole blood) and total radioactivity (whole blood and plasma) at 120, 144, 168, 192, 216, 240, 264, 288, and 312 h post-dose on days 6 to 14 or until discharge. Urine and feces were collected for total radioactivity and metabolite profiling and identification pre-dose and at 24 h intervals post-dose until discharge.

Whole blood samples were assayed for TBP using a validated liquid chromatography tandem mass spectrometry (LC-MS/MS) method (Covance, Madison, WI, USA). The lower limit of quantitation for TBP was <0.0072 mg/mL (15). The TBP blood concentrations were converted to TBP plasma concentrations by correcting for the addition of (1:1) isopropyl alcohol to the collected sample and for plasmatocrit (using an average value of 55%), resulting in a multiplication factor of 3.6. For metabolite profiling, plasma samples were pooled by subject across time points (0.25, 0.5, 1, 2, 4, and 6 h post-dose) to generate 0.25 h to 6 h AUC pools. Urine and feces homogenate samples were pooled by subject to account for greater than 90% of the radioactivity excreted in each matrix. The samples were analyzed via liquid chromatography-high resolution mass spectrometry, with eluent fractions collected into 96-well plates containing solid scintillant at 10 s intervals. The radioactivity in each well was determined using a MicroBeta2 analysis, and radiochemical profiles were generated, based on the radioactivity counts. The quantitation of the metabolites that were present in the plasma, urine, and feces was based on the profiles of radioactivity. The cutoff for the identification of metabolites was 1% of the sample radioactivity for the plasma and 1% of the radioactive dose for the urine and the feces.

### Pharmacokinetic analysis.

The following PK parameters were calculated from the plasma TBP concentrations, blood, and plasma total radioactivity: area under the concentration-time curve from time zero to the last observed nonzero concentration, as calculated by the linear trapezoidal method (AUC_0-last_); area under the concentration-time curve from time zero extrapolated to infinity (AUC_0-inf_); percentage of AUC_0-inf_ extrapolated, represented as (1 − AUC_0-last_/AUC_0-inf_) · 100 · (AUC_%extrap_); last observed (quantifiable) plasma concentration (C_last_); maximum observed concentration (Cmax); time to reach Cmax (T_max_); and apparent first-order terminal elimination half-life. In addition, the ratio of AUC_0-inf_ of the plasma TBP, relative to the AUC_0-inf_ of the plasma total radioactivity, as well as the ratio of the AUC_0-inf_ of the whole blood total radioactivity to the AUC_0-inf_ of the plasma total radioactivity were determined. All of the PK evaluations were performed using Phoenix WinNonlin version 8.1 (Pharsight Corporation, Mountain View, CA, USA).

For the mass balance, the amount excreted in the urine and in the feces, the cumulative amount excreted in the urine and in the feces, the percentage excreted in the urine and in the feces, and the cumulative percentage excreted in the urine and in the feces, relative to the administered dose, were calculated.

### Statistical analysis.

No formal statistical analysis of the PK parameters was performed. The PK population included all of the subjects who received [^14^C]-TBP-PI-HBr and had at least 1 valid bioanalytical result. The safety population included all of the subjects who received [^14^C]-TBP-PI-HBr. The pharmacokinetic concentrations and parameters were summarized using descriptive statistics: N, arithmetic mean, arithmetic standard deviation, coefficient of variation (CV%), geometric mean, geometric CV%, minimum, median, and maximum. All of the statistical evaluations were conducted using SAS version 9.4.

## References

[1] Papp-Wallace K, Endimiani A, Taracila M, Bonomo R. 2011. Carbapenems: past, present, and future. Antimicrob Agents Chemother 55:4943–4960. doi:10.1128/AAC.00296-11. 21859938 PMC3195018

[2] Citron DM, Tyrrell KL, Rubio A, Goldstein EJC. 2018. In vitro activity of tebipenem (SPR859), tebipenem-pivoxil (SPR994) and meropenem against a broad spectrum of anaerobic bacteria. SUN-559. ASM Microbe.

[3] Lacasse E, Brouillette E, Larose A, Parr TR, Jr, Rubio A, Malouin F. 2019. In vitro activity of tebipenem (SPR859) against penicillin-binding proteins of Gram-negative and Gram-positive bacteria. Antimicrob Agents Chemother 63:e02181-18. doi:10.1128/AAC.02181-18. 30718255 PMC6437484

[4] Mendes RE, Rhomberg PR, Huynh H, Cotroneo N, Rubio A, Flamm RK. 2018. Antimicrobial activity of tebipenem (SPR859) against a global challenge set. SUN-558. ASM Microbe 2018. doi:10.1159/000528641. PMC6535533 30936096

[5] Mendes RE, Rhomberg PR, Watters A, Cotroneo N, Rubio A, Flamm RK. 2018. Antimicrobial activity assessment of tebipenem (SPR859) against an isolate collection causing urinary tract infections. ASM Microbe.

[6] Zou Y, Cotroneo N, Rubio A. 2018. In vitro bactericidal activity and post-antibiotic effect of tebipenem (SPR859) against susceptible and extended-spectrum beta-lactamase producing Enterobacteriaceae as compared to levofloxacin (LVX) and meropenem (MEM). SUN-561. ASM Microbe.

[7] Cotroneo N, Rubio A, Critchley IA, Pillar C, Pucci MJ. 2020. In vitro and in vivo characterization of tebipenem, an oral carbapenem. Antimicrob Agents Chemother 64:e02240-19. doi:10.1128/AAC.02240-19. 32423950 PMC7526814

[8] Eckburg PB, Jain A, Walpole S, Moore G, Utley L, Manyak E, Dane A, Melnick D. 2019. Safety, pharmacokinetics, and food effect of tebipenem pivoxil hydrobromide after single and multiple ascending oral doses in healthy adult subjects. Antimicrob Agents Chemother 63:e00618-19. doi:10.1128/AAC.00618-19. 31262768 PMC6709501

[9] Ganesn H, Gupta VK, Safir MC, Bhavani SM, Talley AK, Melnick D, Rubino CM. 2023. Population pharmacokinetic analyses for tebipenem after oral admininistration of prodrug tebipenem pivoxil hydrobromide. Antimicrob Agents Chemother. 10.1128/aac.01451-22 PMC10269146 37191505

[10] Bush K, Bradford P. 2016. β-lactams and β-lactamase inhibitors: an overview. Cold Spring Harb Perspec Med 6:a025247. doi:10.1101/cshperspect.a025247. PMC4968164 27329032

[11] Eckburg PB, Muir L, Critchley IA, Walpole S, Kwak H, Phelan A-M, Moore G, Jain A, Keutzer T, Dane A, Melnick D, Talley AK. 2022. Oral tebipenem pivoxil hydrobromide in complicated urinary tract infection. New Engl J Med 386:1327–1338. doi:10.1056/NEJMoa2105462. 35388666

[12] Kato K, Shirasaka Y, Kuraoka E, Kikuchi A, Iguchi M, Suzuki H, Shibasaki S, Kurosawa T, Tamai I. 2010. Intestinal absorption mechanism of tebipenem pivoxil, a novel oral carbapenem: involvement of human OATP family in apical membrane transport. Mol Pharm 7:1747–1756. doi:10.1021/mp100130b. 20735088

[13] Kijima K, Morita J, Suzuki K, Aoki M, Kato K, Hayashi H, Shibasaki S, Kurosawa T. 2009. Pharmacokinetics of tebipenem pivoxil, a novel oral carbapenem antibiotic, in experimental animals. Jpn J Antibiot 62:214–240. 19882982

[14] Food and Drug Administration. 2016. Guidance for industry: safety testing of drug metabolites (revision 1). Available from: http://sp2013.ent.covance.com/sites/LSDS/MedSci/Global_Reg_Affairs/Reg_Writing/TrainingMaterials/EarlyClinicalServices/CovanceInternal/StudyDesignPrinciples/MetaboliteProfiling.

[15] International Conference on Harmonisation. 2012. Harmonised tripartite guideline: guidance on nonclinical safety studies for the conduct of human clinical trials and marketing authorization for pharmaceuticals M3(R2) questions and answers (R2). Available from: http://www.ich.org/fileadmin/Public_Web_Site/ICH_Products/Guidelines/Efficacy/E1/Step4/E1_Guideline.pdf. 20349552

